# Self-rated health among Greenlandic Inuit and Norwegian Sami adolescents: associated risk and protective correlates

**DOI:** 10.3402/ijch.v72i0.19793

**Published:** 2013-02-06

**Authors:** Anna Rita Spein, Cecilia Petrine Pedersen, Anne Cathrine Silviken, Marita Melhus, Siv Eli Kvernmo, Peter Bjerregaard

**Affiliations:** 1 Centre for Sami Health Research, Faculty of Health Sciences, University of Tromsø, Karasjok, Norway; 2 Center for Health Research in Greenland, National Institute of Public Health, University of Southern Denmark, Copenhagen K, Denmark; 3 Department of Community Medicine, Centre for Sami Health Research, Faculty of Health Sciences, University of Tromsø, Tromsø, Norway; 4 Department of Child and Adolescent Psychiatry, Division of Child and Adolescent Health, University Hospital of North Norway, Tromsø, Norway; 5 Department of Community Medicine, Faculty of Health Sciences, University of Tromsø, Tromsø, Norway

**Keywords:** adolescents, Arctic, indigenous, Inuit, protective factors, risk factors, Sami, self-rated health (SRH), suicidal behaviours

## Abstract

**Objectives:**

Self-rated health (SRH) and associated risk and protective correlates were investigated among two indigenous adolescent populations, Greenlandic Inuit and Norwegian Sami.

**Design:**

Cross-sectional data were collected from “Well-being among Youth in Greenland” (WBYG) and “The Norwegian Arctic Adolescent Health Study” (NAAHS), conducted during 2003–2005 and comprising 10th and 11th graders, 378 Inuit and 350 Sami.

**Methods:**

SRH was assessed by one single item, using a 4-point and 5-point scale for NAAHS and WBYG, respectively. Logistic regressions were performed separately for each indigenous group using a dichotomous measure with “very good” (NAAHS) and “very good/good” (WBYG) as reference categories. We simultaneously controlled for various socio-demographics, risk correlates (drinking, smoking, violence and suicidal behaviour) and protective correlates (physical activity, well-being in school, number of close friends and adolescent–parent relationship).

**Results:**

A majority of both Inuit (62%) and Sami (89%) youth reported “good” or “very good” SRH. The proportion of “poor/fair/not so good” SRH was three times higher among Inuit than Sami (38% vs. 11%, p≤0.001). Significantly more Inuit females than males reported “poor/fair” SRH (44% vs. 29%, p≤0.05), while no gender differences occurred among Sami (12% vs. 9%, p≤0.08). In both indigenous groups, suicidal thoughts (risk) and physical activity (protective) were associated with poor and good SRH, respectively.

**Conclusions:**

In accordance with other studies of indigenous adolescents, suicidal thoughts were strongly associated with poorer SRH among Sami and Inuit. The Inuit–Sami differences in SRH could partly be due to higher “risk” and lower “protective” correlates among Inuit than Sami. The positive impact of physical activity on SRH needs to be targeted in future intervention programs.

Self-rated health (SRH) has been widely used as a measure of current health status both among and within ethnic groups and nationalities. Across ages, ethnic minorities and indigenous people generally rate their health worse than majority peers (1). SRH is found to be an important predictor of mortality, morbidity, health service use, medical use and general well-being (2–5). Studies have revealed several correlates associated with SRH (6), including demographics (e.g. gender, ethnicity), educational factors (e.g. parental education), economic factors (e.g. self-rated economic position), geographical aspects (e.g. urban–rural), various social correlates (e.g. marital status, family relationship, violence and sexual abuse) and also health-related behaviours (e.g. smoking, drinking and physical activity). Longitudinal studies have revealed that SRH is quite a stable construct during adolescence (2).

Little research has been conducted on SRH among indigenous adolescents (1, 7, 8). Bombak and Bruce (1) in their review of SRH and ethnicity found that many of the factors generally associated with poorer SRH have been replicated among North American indigenous youth, including females, poor family income, poor body image and weight concerns, physical and sexual abuse, suicide attempts, substance abuse and nutritional inadequacies. In contrast, social competence, good school performance, non-smoking and having recently had a physical examination were positively associated with SRH (1). Among 11-to-17-year-old Greenlanders (majority Greenlandic born Inuit), older students reported their health more negatively than younger peers (7), with a greater likelihood of poor SRH for both youths who were bullied and those who bullied others (8).

In Norway, the Sami people number about 60,000, and their current status is that of an indigenous people. With the exception of the Sami Highland in Finnmark County, the Sami is a numeric minority. The colonisation of Sapmi (land of the Sami people) and the former assimilation policy called “Norwegianization“ has contributed to loss of ethnic identity and language shift among many Sami, although in some geographical regions this impact has been less severe. During the last decades, a political shift towards integration has contributed to increased ethnic awareness and revitalisation about Sami cultural, linguistic, social, health, politically issues etc., and has also contributed to the establishment of Sami institutions within media, school, health services, art, as well as the Sami parliament in 1989 (9).

Greenland is the world's largest island with a total population numbering only about 57,000 of whom 90% are Inuit. Greenland was colonised by Denmark in 1721 and reforms of the Greenlandic infrastructure, industry and welfare during the 1950s and 60s were based on Danish administrative systems. The profound changes on everyday life and in the social structures of the communities have accelerated since 1950 and most Greenlanders have experienced the transition during their own life time. In 1979 Greenland achieved Home Rule and in 2009, Self-Government, the last step before complete independence. In the process of economic, social and cultural change, the most central changes were a shift from subsistence hunting and fishing to wage earning; population movement from small villages to larger towns; and increased availability of formal education, accompanied by changing life style and eventually by changes in health status. Today the isolated populations in villages and smaller towns face the largest challenges with limited opportunities. In contrast, life in the capital reflects a contemporary Scandinavian lifestyle with a wide range of educational, occupational and recreational possibilities. Greenlandic is the dominant language, but Danish is used in higher education (9).

A variety of studies have investigated health behaviour among Norwegian Sami and Greenlandic Inuit adolescents. Among young Sami, a wide range of health compromising behaviours including emotional and behavioural problems, suicidal behaviour, substance use and their relationship with ethnocultural factors have been studied (9–13). Generally, no differences have been found with regard to smoking, suicidal behaviour and general behavioural/conduct problems, while higher abstinence and lower drinking rates have been shown to occur among young Sami compared to the majority of Norwegians (9, 12, 13). A review of child and adolescent health in Greenland, primarily among Greenlandic born Inuit, concluded that negative health behaviour such as smoking and binge drinking, but also obesity, poor oral health, unhealthy diet and sexual risk behaviour frequently occurred (14). In their review, Lethi and colleagues (15) noted more suicidal problems among young Greenlandic Inuit than their Sami peers.

The impact of social change on health is more transparent in circumpolar populations and regions where social changes have occurred more recently, rapidly and dramatically. The Sami and Greenlandic Inuit adolescents are both a product of and are growing up in societies with a common history of colonisation and assimilation. In Greenland, these changes have contributed to increasing alcohol dependency, youth suicides and violence (9, 16). Among Sami youth, assimilation and weak ethnic identity have been associated with more substance use (12). Traditional indigenous lifestyles such as reindeer herding among Sami have been affected by increasing work-related stress including suicidal behaviour problems (12, 17). Nonetheless, the colonised history and the current political and economic situation are different in many ways as, for example, the Sami population is a minority in Norway, while the Greenlandic Inuit population is a majority still connected legally to the Danish state. These similarities and differences make it important to compare these two adolescent groups and how the social change has made an impact on their health and well-being. From a public health perspective, it is very important to understand and increase the knowledge of SRH among the two indigenous adolescent groups, the Sami and Inuit, as SRH is a measure of great predictive value for future poor health and death (2, 18).

In this paper, we first examine whether Sami and Inuit adolescents rate their health differently and compare this to regional and national figures. Second, we examine gender differences in SRH for the two indigenous groups, but also with regard to ethnic differences separately for males and females. Third, we investigate the correlates associated with poor versus good SRH for Sami and Inuit separately. In accordance with existing research on adolescent SRH (1, 2, 6, 19), we included four potential risk factors (drinking, smoking, suicidal behaviour and experienced violence) and four protective factors (physical activity, well-being in school, number of friends and adolescent–parent relationship) and simultaneously controlled for various socio-demographics.

## Material and methods

### Study samples

Cross-sectional data from two school-based studies: “The Norwegian Arctic Adolescent Health Study” (NAAHS) and “Well-being among Youth in Greenland” (WBYG) were used. The NAAHS was a study on health and living conditions among indigenous Sami and non-Sami adolescents in rural and semi-rural areas and in towns in the three northernmost counties of Norway. The questionnaire included somatic health complaints, medicine use and health services uses, food habits, physical activity, schooling and educational plans, cultural activities and traditions, sexual behaviour, as well as broader mental health issues and risk-taking behaviours including self-efficacy, stress and coping, anxiety and depression, substance use, self-harm and suicidal behaviour, bullying and sexual abuse. All 293 junior high schools in North-Norway were invited to participate in the study, of which only one school refused to take part in the study. The percentage of Sami students varied between schools with the highest number in the Sami dominated areas of Finnmark.

The WBYG was a study on well-being, health behaviour and health among adolescents in Greenland. The WBYG included socio-demographic factors, family and upbringing conditions, social relations, school and leisure time factors, health behaviour, physical and mental health with a special focus on suicidal behaviour, sexual behaviour and sexual abuse. In the WBYG, students from 10 schools above the age of 14 in grade 10 and 11 were invited to participate in the study. The selected schools represented all of the different geographical areas in Greenland—North, South, West and East. Moreover, school size and the number of adolescents from villages was taken into account so that towns with large schools were ideal for getting as many participants as possible (20).

Table I summarises the two working samples, study periods and participation rates. A paper (NAAHS) and electronic (WBYG) bilingual questionnaire was available in both studies (North Sami dialect and Norwegian in NAAHS and Greenlandic and Danish in WBYG) (10, 13, 20). The WBYG included overall 508 10th and 11th grade students. Here, only a subsample of 378 10th (N=107) and 11th (N=271) graders was selected for reason of age (15–16 year olds) comparison with NAAHS. The school absentee rate for the whole WBYG sample of all 10th to 11th graders was 25% and was similar to the absentee rate caused by illness within the last 2 weeks prior to the study (22%), which is the same for this subsample. No data on the overall school absentee rate was available in the NAAHS. The subsample of indigenous Sami included 358 adolescents.

**Table I:** Study descriptions: The Norwegian Arctic Adolescent Health Study (NAAHS) and Well-Being among Youth in Greenland (WBYG)

	Sami (NAAHS)	Inuit (WBYG)
Study year	2003–05	2004–05
Invited (n)^a^	5877	663
Participants (n)	4880	508
Response rate (%)	83	75
Number of participating schools (n)	292^b^	10
Working sample of 15–16-year olds^c^	350	378

a Including both indigenous Sami and Greenlandic Inuit, but also Norwegian and Danish adolescents.

b At 116 junior high schools one or more students reported Sami origin.

c NAAHS: 10th graders (98% were 16 year olds); WBYG: 10th and 11th graders.

### Instruments

#### Ethnicity

In the NAAHS eight “objective” questions tapped ethnicity; father's and mother's ethnicity and spoken language by each of the parents and grandparents, separately. All questions had answering options as Norwegian, Sami, Kven, Finnish and “Other”, and multiple answers were allowed. These questions were combined into ethnic background where respondents were categorised as Sami if they had answered Sami on one or more of these questions. In addition, there were “subjective” questions about the respondent's own ethnicity and five questions on how they view themselves: *I regard myself Norwegian/Sami/Kven/Finnish/Other*. For each of the five ethnic groups, respondents were able to answer on a 4-point scale, ranging from “fully agree” to “fully disagree”. There were 91 people who stated that they fully or partly agreed that they regarded themselves Sami or having Sami ethnicity, without reporting Sami ethnicity or language for any of the parents or grandparents. We decided to use only objective criteria to identify Sami youth to avoid misclassification of youth with very weak or no Sami affiliation.

In the WBYG, Inuit background was based on the question: *Would you describe yourself as a Greenlander or a Dane*? We excluded those defining themselves as *only Danish* (n=12), *Something else* (n=4) and *I don't know* (n=16).

#### Self-rated health

In the NAAHS, SRH was based on one question: *What is your present state of health?* with reply alternatives: *poor*, *not so good*, *good* and *very good*. In the WBYG, SHR was based on: *How would you rate your health?* with five reply alternatives: *very poor, poor, fair, good* and *very good*. However, no Inuit participants reported the fifth category *very poor*. In the logistic regression analyses, SRH was dichotomised into: (a) Poor (*fair/poor*) and (b) Good (*very good/good*) in WBYG. In NAAHS, SRH was categorised into: (a) Poor (*good/not so good/poor*) and (b) Good (*very good*). In NAAHS and WBYG, there were 9 and 11 missing values on the SRH variables, respectively. The reason for choosing different cut-offs for SRH in NAAHS and WBYG was that the distribution of SRH varied considerably between the two study samples. There were few observations in the *very good*, and none in the *very poor* category among Inuit, and few in the *not so good* and *poor* category among Sami.

Table II shows the independent socio-demographic, risk and protective variables in NAAHS and WBYG. Also presented are the items included, their categories and final measurements.

**Table II:** Description of the independent variables in NAAHS and WBYG

Variables	Sami (NAAHS)		Inuit (WBYG)		NAAHS/WBYG
		
	Item(s)	Categories	Item(s)	Categories	Measurement(s)
**Socio-demographics**					
Socio-economic status	*I think that our family, seen in relation to other families in Norway, have:*	Poor economy Average economy Good economy Very good economy	*What is your mother's (father's) formal education?*	Elementary school High school Short vocational education Higher education, 3–4 years University education	**NAAHS**: **Good** (good, very good economy) vs. **Poor** (poor, average economy) **WBYG: Higher** educational level (college, university degree) vs. **Lower** educational level (elementary school, high school, short vocational education)
Parents married/cohabiting	*My parents are?*	Married/cohabiting Unmarried Divorced/separated One or both are dead Other	*Do your parents live together?*	Yes No	**Yes** (married/cohabiting/ living together) vs. **No** (unmarried, divorced/ separated, dead, other, or not living together)
**Risk correlates**					
Suicidal behaviour	*Have you ever had suicidal thoughts?*	No Yes	*Have you ever seriously considered committing suicide?*	No Yes	**No** vs. **Suicidal thoughts** (yes) vs. **Suicidal attempts** (Yes on suicidal thoughts and/or attempts)
	*Have you ever had suicidal attempts?*	No Yes	*Have you ever attempted to commit suicide?*	No Yes	
Alcohol use	*Have you ever drunk alcohol?*	No Yes	*How often do you drink alcohol?*	I never drink alcohol Less than monthly About once a month About once weekly Daily or almost daily	**Abstinence** (Never drink alcohol or not drinking during the last year) vs. **Moderate** (Drinking monthly or less) vs. **Frequent** (Drinking once weekly or more)
	*About how often in the course of the past year have you drunk alcohol?*	4–7 times weekly 2–3 times weekly About once a week 2–3 times monthly About once a month A few times during the last year			
		Have not drunk alcohol during the last year			
Current smoking	*Do you smoke, or have you smoked?*	No, never Yes, but I quit Yes, occasionally Yes, daily	*Do you smoke?*	No Yes, but I have days where I don‘t smoke Yes, daily	**No** (Never or non-smokers, and quitters) vs. **Yes** (Occasional smokers and daily smokers)
Experienced violence	*Have you yourself been exposed to violence (been hit, kicked, or similar) during the last 12 months?*	Never Yes, only by youth Yes, only by adults Yes, both youth and adults	* Within the last year have you been exposed to any of the following?^a^* *Most children experience conflicts at home. Have you experienced* *- You were pushed and shaken in anger?* - *You were torn in your hair* *You were beaten*	Yes Yes	**No** (Never, not exposed to violence within the last year, and not exposed to domestic violence (e.g. conflicts at home)) vs. **Yes** (Exposed to violence within the last year and/or domestic violence (conflicts at home)
**Protective correlates**					
Physical activity	*Out of school hours: how many times per week do you take part in sport/do physical exercise to the extent that you get out of breath or sweat?*	0,1,2,3,4,5,….	*How often do you exercise hard (running, soccer, something else)*	Every day At least once a week Less than weekly Never	**Frequent** (1 or more times a week or every day) vs. **Seldom** (0 times a week, less than weekly or never)
Well-being in school	*Do you enjoy going to school*?	Fully agree Partly agree Partly disagree Fully disagree	*Do you like going to school?*	Very well Well Fair Not so well	**Good** (Fully or partly agree, well and very well) vs. **Poor** (Partly or fully disagree, fair or not so well)
Number of friends	*About how many friends do you have (do not include siblings)*	None 1 2–3 4 or more	*How many close friends do you have?*	No close friends at the moment 1 2–3 4 or more	**Few** (None or 1) vs. **Many** (2 or more)
Adolescent–parent relationship	*If you have personal problems who could you go to*?^b^	Yes No I don‘t know	*How easy or difficult is it for you to talk with the following persons, when you have problems?^b^*	Very easy Easy Difficult Very difficult I don‘t have any parents^c^	**Good** (yes, very easy, easy) vs. **Poor** (no, difficult, very difficult)
					

a Violence was one of ten alternatives.

b Parents were one of several alternatives.

c The alternative “I don’t have any parents” was categorized as missing.

### Statistical analysis

To evaluate both Sami–Inuit and in-group differences in the distribution of covariates, Pearson's Chi-squared test or Fischer's exact test were used for categorical data. The cells contributing most to the statistical differences were determined by inspection of the standardised residuals. Logistic regressions were performed with SRH as the binary dependent variable, as in the majority (68%) of studies of youth SRH (6). Associations between SRH and each risk, protective and socio-demographic factors were explored by simple logistic regressions, and separately for Sami and Inuit. In line with the majority of studies of SRH (91%), we used multiple models (6) to examine the influence of each socio-demographic, risk and protective variable when mutually controlling for all other factors. A stepwise backward approach was chosen. Starting with a full model, the variable with the highest p-value was removed, and the procedure was repeated until all remaining variables were significant at the 10% level. Regardless of the significant level at each step, gender was forced into the model due to the possibility of causing any confusion. SPSS version 19 was used for statistical analysis.

## Results

### SRH by indigenous group and gender

Table III shows that the overall proportion of Sami (N=350) and Inuit (N=378) adolescents reported *poor*, *not so good/fair*, *good* and *very good* SRH. *Poor* and *not so good/fair* SRH were three times higher among Inuit than Sami. Significant differences in SRH occurred among Sami and Inuit, as fewer Sami youth reported *not so good* SRH, while more Inuit report *fair* SRH (p≤0.001). Significant ethnic differences in SRH occurred for both males and females. These differences were due to fewer Sami reporting *not so good*/*fair* SRH when compared to Inuit counterparts; the figures were 11% vs. 41% for females [***p≤0.001] and 9% vs. 28% for males [***p≤0.001]. Significant ethnic differences in fair SRH occurred for both males and females as fewer Sami youth reported fair SRH than their Inuit counterparts. Significant gender differences in SRH were only found for Inuit (*p≤0.05), but not for Sami (p≤0.08, Table III).

**Table III:** Distribution of self-rated health in NAAHS and WBYG by gender

	Sami^a^ (NAAHS)			Inuit^a^ (WBYG)		
	
	Male n=162	Female n=188	Total^b^ N=350	Male n=155	Female n=223	Total^c^ N=378
Self-rated health						
Very good (n, %)	64 (40)	54 (29)	118 (34)	32 (21)	32 (14)	64 (17)
Good (n, %)	83 (51)	111 (59)	194 (55)	78 (50)	93 (42)	171 (45)
Fair/Not so good (n, %)	15 (9)	20 (11)	35 (10)	44 (28)	91 (41)	135 (36)
Poor (n, %)	0 (0)	3 (2)	3 (1)	1 (1)	7 (3)	8 (2)

a Significant Sami–Inuit differences in SRH, disfavouring Inuit [χ^2^ (3, 728)=77.6, ***p≤0.001], due to fewer Sami reporting *not so good* SRH and more Inuit reporting *fair* SRH.

b Non-significant gender differences in SRH among Sami [χ^2^ (3, 350)=6.71, p≤0.08].

c Significant gender differences in SRH among Inuit [χ^2^ (3, 378)=10.14, *p≤0.05].

### Distribution of socio-demographics, risk and protective correlates by indigenous group

Table IV shows the distribution of the independent variables in Sami and Inuit adolescents. There were significant Sami–Inuit group differences for all risk and protective correlates, except for reporting of experienced violence, which occurred in about a quarter of the samples. Overall, suicidal attempts were reported twice as often by Inuit than by Sami (Table IV). Inuit adolescents found it significantly easier to talk with their parents than Sami, while significantly more Sami than Inuit reported good well-being in school. A significantly higher proportion of Sami than Inuit also reported frequent physical activity. While significantly more young Inuit were current smokers, more Inuit than Sami abstained from alcohol (Table IV).

**Table IV:** The distribution of independent variables in NAAHS and WBYG by gender (n, %)

		Sami (NAAHS)				Inuit (WBYG)				
	
		Male n=162	Female n=188	Total N=350	Effect of gender – Sami (*p*)	Male n=155	Female n=223	Total N=378	Effect of gender –Inuit (*p*)	Effect of indigenous group (*p*)
**Socio-demographics**										
Parents married/ cohabiting	Yes	88 (55)	117 (62)	205 (59)	0.15	93 (60)	130 (59)	223 (59)	0.75^a^	0.88
	No	73 (45)	71 (38)	144 (41)		61 (40)	92 (41)	153 (41)		
Socio-economic status	Good	102 (65)	95 (51)	197 (57)	0.01	101 (67)	140 (66)	241 (67)	0.82^a^	NT^b^
	Poor	56 (35)	92 (49)	148 (43)		49 (33)	72 (34)	121 (33)		
**Risk correlates**										
Suicidal behaviour	No	119 (74)	91 (49)	210 (61)	≤0.001	126 (82)	98 (44)	224 (60)	≤0.001	≤0.001
	Thoughts	31 (19)	67 (36)	98 (28)		15 (10)	50 (23)	65 (17)		
	Attempts^c^	10 (6)	27 (15)	37 (11)		13 (8)	73 (33)	86 (23)		
Alcohol use	Abstinence	20 (14)	12 (8)	32 (11)	0.20	32 (21)	35 (16)	67 (18)	0.25	0.03
	Moderate	103 (74)	122 (80)	225 (77)		105 (68)	153 (69)	258 (68)		
	Frequent	16 (12)	19 (12)	35 (12)		17 (11)	35 (16)	52 (14)		
Current smoking	No	110 (68)	120 (64)	230 (66)	0.46	85 (55)	90 (40)	175 (46)	0.005^a^	≤0.001
	Yes	52 (32)	67 (36)	119 (34)		69 (44)	133 (60)	202 (54)		
Experienced violence	No	118 (74)	154 (82)	272 (78)	0.05	128 (83)	176 (79)	304 (80)	0.23^a^	0.50
	Yes	42 (26)	33 (18)	75 (22)		27 (17)	47 (21)	74 (20)		
**Protective correlates**										
Physical activity	Frequent	144 (90)	154 (85)	298 (87)	0.17	106 (70)	105 (47)	211 (56)	≤0.001^a^	≤0.001
	Seldom	16 (10)	27 (15)	43 (13)		46 (30)	117 (53)	163 (44)		
Well-being in school	Good	142 (88)	170 (90)	312 (89)	0.50	115 (75)	170 (78)	285 (77)	0.62^a^	≤0.001
	Poor	19 (12)	18 (10)	37 (11)		38 (25)	49 (22)	87 (23)		
Number of close friends	2 or more	153 (95)	177 (95)	330 (95)	0.87	142 (92)	193 (88)	335 (90)	0.23	0.01
	0 or one friend	8 (5)	10 (5)	18 (5)		12 (8)	26 (12)	38 (10)		
Adolescent–parent relationship	Good	85 (56)	96 (52)	181 (54)	0.42	104 (78)	138 (67)	242 (71)	0.03	≤0.001
	Poor	66 (44)	89 (48)	155 (46)		29 (22)	68 (33)	97 (29)		

a Fisher Exact Test used. There were no significant differences in the gender distribution in NAAHS and WBYG samples; [χ^2^ (1, n=728)=2.06, NS].

b NT: Not Tested. Indigenous group differences were not tested due to different socio-economic measures in NAAHS (parental financial situation) and WBYG (parental educational level).

c Includes both suicidal attempts and suicidal thoughts. The number reporting only prior suicidal attempts and not thoughts were 15 in total (4 Sami/11 Inuit). The three categories in the suicidal behaviour item are mutually exclusive.

### Distribution of socio-demographics, risk and protective correlates by gender

Table IV shows that among both Sami and Inuit suicidal thoughts and attempts were reported significantly more often by females than males. Significantly more Sami males had experienced violence during the last year, while females reported significantly more poor parental economy. Significantly more Inuit females than males were smokers, while Inuit females reported more difficulty in talking with their parents than males (Table IV). Inuit females were also significantly less physically active; slightly more than half of them reported seldom engaging in physical activity compared to about one third of their male counterparts (Table IV).

### Logistic regression analyses: socio-demographics, risk and protective correlates associated with SRH in Sami (NAAHS) and Inuit (WBYG)

Table V shows the simple and multiple logistic regression analyses of correlates associated with SRH among Sami and Inuit adolescents separately. For both groups, adjusted analyses revealed that suicidal thoughts and physical inactivity were risk factors for poor SRH. Suicidal thoughts increased the risk of poor SRH by almost four times among Sami and by about two times among Inuit. In contrast, physical activity decreased the risk of poor SRH by almost five times among Sami and two times among Inuit. Among Sami unadjusted risk correlates of poor SRH also included perception of poor family economy, experience of violence, heavy drinking and poor well-being in school. In the final multiple regression analysis, only poor parental economy was associated with poor SRH. Among Inuit, with the exception of gender, an additional two correlates remained significant in the final multiple regression analysis. Being a current smoker increased the risk of poor SRH twofold, while a good parent–adolescent relationship decreased the risk almost twofold. Number of friends was not related to SRH in unadjusted or adjusted analysis.

**Table V:** Simple and multiple logistic regressions of poor vs. good self-rated health in NAAHS and WBYG

		Sami (NAAHS)				Inuit (WBYG)			
	
		Unadjusted (n=350)		Adjusted^a^ (n=265)^b^		Unadjusted (n=378)		Adjusted^a^ (n=333)^b^	
			
		OR	95% CI	OR	95% CI	OR	95% CI	OR	95% CI
**Socio-demographics**									
Parents married/ cohabiting	Yes	1	Ref.			1	Ref.		
	No	1.56	(0.99,2.48)^NS^			1.04	(0.68,1.59)^NS^		
Socio-economic status^c^	Good	1	Ref.	1	Ref	1	Ref.		
	Poor	2.81	(1.73,4.56)***	2.74	(1.60,4.69)***	0.73	(0.46,1.15)^NS^		
Gender	Male	1	Ref.	1	Ref.	1	Ref.	1	Ref.
	Female	1.62	(1.04,2.53)*	1.08	(0.65,1.82)^NS^	1.92	(1.24,2.96)**	1.08	(0.63,1.84)^NS^
**Risk correlates**									
Suicidal behaviour	No	1	Ref.	1	Ref.	1	Ref.	1	Ref.
	Thoughts	4.57	(2.48,8.44)***	3.77	(1.96,7.25)***	2.74	(1.56,4.83)***	2.07	(1.10,3.91)***
	Attempts^d^	4.27	(1.71,10.7)***	2.64	(0.98,7.08)^NS^	2.50	(1.50,4.17)***	1.66	(0.87,3.18)^NS^
Alcohol use	Abstinence	1	Ref.			1	Ref.		
	Moderate	1.50	(0.71,3.17)^NS^			1.71	(0.95,3.07)^NS^		
	Frequent	4.67	(1.44,15.1)**			1.58	(0.73,3.41)^NS^		
Current smoking	No	1	Ref.	1	Ref.	1	Ref.	1	Ref.
	Yes	2.39	(1.43,3.97)***	1.64	(0.92,2.91)^NS^	2.72	(1.76,4.22)***	2.05	(1.23,3.40)**
Experienced violence	No	1	Ref.			1	Ref.		
	Yes	1.81	(1.01,3.25)*			1.07	(0.64,1.81)^NS^		
**Protective correlates**									
Physical activity	Seldom	1	Ref.	1	Ref.	1	Ref.	1	Ref.
	Frequent	0.17	(0.06,0.50)***	0.21	(0.07,0.63)**	0.41	(027,0.63)***	0.50	(0.31,0.81)**
Well-being in school	Poor	1	Ref.	1	Ref.				
	Good	0.35	(0.14,0.87)*	0.39	(0.14,1.11)^NS^				
Adolescent–parent relationship	Poor	1	Ref.	1	Ref.	1	Ref.	1	Ref.
	Good	0.68	(0.43,1.08)^NS^	0.39	(0.75,2.68)^NS^	0.46	(0.29,0.75)*	0.59	(0.35,0.99)*
Number of friends^e^	0–1 friend	1	Ref.			1	Ref.		
	≥2 friends	1.03	(0.38,2.81)^NS^			1.40	(0.71,2.75)^NS^		

Ref: reference group; OR: odds ratio; CI: confidence interval

*** p≤0.001

** p≤0.01

* p≤0.05

NS non-significant.

a Each independent variable was adjusted for all other socio-demographics, risk- and protective correlates.

b Number of cases included in the final backward logistic regression. The significance level for the variables included was set at 10%.

c Socio-economic measures by parental financial situation in NAAHS and by parental educational level in WBYG.

d Includes both suicidal attempts and suicidal thoughts. The number reporting only prior suicidal attempts and not thoughts were 15 in total (4 Sami/11 Inuit). The three categories in the suicidal behaviour item are mutually exclusive.

e Number of friends was not included in the multivariate analyses.

## Discussion

This paper describes patterns of SRH among Inuit and Sami adolescents. The majority of young Sami and Inuit reported *good* or *very good* SRH. Very few reported *poor* health. The NAAHS is the first study to investigate SRH among Sami adolescents, while SRH has previously been assessed among Greenlandic youth (7, 8). Our first aim was to compare rates of SRH among Sami and Inuit to regional and national peers, as well as to each other. The proportions of Sami youth reporting *good* or *very good* SRH (89%) and *poor* SRH (11%) in the NAAHS do not differ greatly from regional (Non-Sami in the NAAHS, results not shown) or national peers (2). However, this finding contrasts with data on adult Sami who have reported poorer SRH than Norwegian peers (21). Among adult Sami, poor SRH was associated with older age, female gender and lower educational and income level, but became non-significant when controlling for discrimination (21). The differences in SRH among Sami adolescents and adults (≥51 year) may be attributable to the earlier “Norwegianization” policy (e.g. institutional discrimination) that older age groups have experienced. Today Sami women living in Norwegian dominated areas (with greater integration and assimilation) that are characterised by less ethnic support reported the most unsatisfactory conditions concerning SRH (21). The 15–17 year olds in the WBYG showed lower rates for *good* and *very good* health when compared to findings from the Greenlandic Health Behaviour in School-aged Children (HBSC) study (2006; 6th to 11th graders); the figures were 71% (80%) and 64% (66%) for 15-year-old males and females, respectively (7). Cross-cultural data from the Scandinavian part of the HBSC study in 2005/2006, using a 4-point SRH scale, found the highest rate of *poor* and *fair* SRH in Greenland (~21%), followed by Norway (~19%), then Denmark (~14%) (22).

Although most adolescents reported good SRH, there was a relatively large difference in how Inuit and Sami perceived their health. Twice as many Sami as Inuit reported *very good* SRH (34% vs. 17%). More than two thirds (36%) of Inuit reported their health to be *fair*, while the corresponding *not so good* among Sami was 10%. These findings may partly represent significant Sami–Inuit differences in risk (suicidal behaviour and smoking disfavouring Inuit) and protective correlates (Sami being more physically active). Nonetheless, the majority of indigenous youth did not face significant health risks. Only the prevalence of experiencing violence was similar among Sami and Inuit. However, violence was not significantly associated with poor SRH among Sami in the adjusted analysis, in contrast to findings by others (23). Half of the Inuit adolescents currently smoked and smokers had a more than twofold higher risk of poor SRH than non-smokers. Smoking *per se* may affect SRH either by causing physical health problems, or being used as self-medication for mental stressors (24, 25). There are three main possible explanatory factors for the higher smoking rates found among Inuit than Sami adolescents. First, Sami adolescents are less exposed to environmental smoking as smoking rates of adult Sami – the “parental” generation – tend to be much lower than among Greenlanders (9). Second, passive smoking is less accepted in Norway as the tobacco control policy is more extensive than in Greenland and the Norwegian efforts has also been considered less liberal in its design. For example, a smoking ban was implemented much earlier in Norway (1st of June 2004) compared to Greenland (1st of October 2010) (26). Third, smoking is highly associated with levels of education, which is currently higher among Sami than Inuit peers, as about 39% and 21% of Sami (people in Sami areas) and Greenlanders have attained high school diplomas, respectively (27, 28).

An earlier review concluded that substance use prevalence was not comparable among young Sami and Inuit (15). Moderate drinking may be considered to be normal behaviour as more than three fourths of indigenous Sami and Inuit adolescents responded positively. Although drinking is an inappropriate behaviour in socio-cultural–religious terms among many Sami due to the strong anti-alcohol stand of Læstadianism (9), more Inuit than Sami youth (18% vs. 11%) reported abstinence. Both Sami and Inuit adolescents have generally showed higher abstinence rates than their Norwegian and Danish peers, respectively (9, 29). Frequent drinking was reported by 12% of Sami, but was not significantly associated with poor SRH in the adjusted analysis. However, drinking style was not assessed. The Greenlandic drinking style is characterised by binge drinking, which is less common in Sami adolescents than non-Sami (9, 29).

The second aim of the study was to describe gender differences in SRH. Although males more frequently reported good SRH than females, the crude data showed only significant gender differences in SRH among Inuit. However, gender was not a significant predictor of SRH among Inuit in the final multiple regression analysis. This finding suggests that gender differences in SRH may have been mediated by other socio-demographic, risk and protective factors, in line with the existing literature (2). Among Circumpolar indigenous adults, minimal gender differences in SRH have been found, while female gender has been negatively associated with SRH among Native American youth (1).

Our third aim was to examine ethnic differences and similarities in risk and protective correlates associated with SRH. Of particular note is the almost threefold negative influence of poor socio-economic status on SRH among Sami. Generally, the Sami settlements are characterised by 10% and 18% lower mean income, when compared to regional and national figures, respectively (30). Higher income has been positively associated with SRH among indigenous adults (1, 9). Income seems to be a more relevant factor to adolescents’ health perception than parental education and occupation (19). Young people's subjective perception of poor familial socio-economic status (SES) has been associated with poor SRH even when controlling for race, parental education and total household income (31). In the NAAHS, parental financial status was based on young people's self-reported perception, while the WBYG assessed parental education. This may explain the different impact of SES on SRH among the two indigenous groups.

Our main focus for the remaining discussion will be on the relationship of SRH to suicidal behaviour and physical activity, as correlations were found in both cases among Sami and Inuit. Suicidal thoughts were a strong risk correlate, increasing the odds for poor SRH twofold (Inuit) and fourfold (Sami). Suicide is a huge health problem in Arctic communities. Suicide is the leading cause of death among 15–24 year old indigenous males (9, 16). From 1970 to 1998, suicide rates among young Sami males and females were respectively 53 and 16 per 100,000 person-years, giving a male–female ratio of 3.5:1 (9). In the Sami dominated area of inner Finnmark County there has been a cluster of suicide (9). In Greenland during 1990–99, suicides among 15–24 year olds amounted to 400–500 for men and 100–150 for women per 100,000 person-years, with a male–female ratio of 4.3:1 (16). Suicide seems to be less frequent among young Sami than young Inuit (15). This Norwegian–Greenlandic pattern has also been found with regard to suicidal thoughts among females in the two youngest age groups (14–34 year olds) in the recent “Survey of living conditions in the Arctic” (SLICA) study. This difference was thought to be due to differences in national educational level (32).

Furthermore, Grossman, Milligan and Deyo (33) found in a sample of Native American adolescents that 15% reported a prior suicide attempt and that poor self-perception of health was one of several risk factors for suicide attempts. Among Sami adolescents, risk factors diverging from traditional Sami cultural norms were associated with suicide attempts, including alcohol intoxication, single-parent households and paternal overprotection (9). Suicide of a close friend and loneliness were associated with suicidal behaviour among Greenlandic Inuit adolescents. Having experienced a sexual assault and problems with parents were female-specific factors, while termination of a relationship was specifically associated with Inuit male suicidal behaviour (9). Suicidal thoughts were more frequently reported among young Inuit (in particular females) from homes with poor emotional environments, alcohol problems and violence (16).

Engaging in frequent physical activity decreased the odds of poor SRH about twofold (Inuit) and fivefold (Sami). The NAAHS survey referred to physical exercise outside school but no such distinction was made in the WBYG. In line with most studies, there were significant gender differences among Inuit only, adolescent males being more physically active than female peers (9, 34, 35). The differences in physical activity level among the Inuit and Sami can partly be explained by the different measures and a lower access to active leisure time offers in Greenland compared to Norway. Physical activity also positively influences self-image, family and peer relationships and general well-being among youth and has been inversely related to youth depression (35, 36). Studies among indigenous adult groups have revealed lower leisure time physical activity when compared to majority peers, and that lower education and income are negatively associated with physical activity (9). Sami adults seem to be more physically active at work than non-Sami (9).

Both poor SRH *per se*, and the Sami–Inuit differences in SRH may partly be caused by other factors outside this study that contribute to inequalities in SRH. Considerable differences in health indicators exist within the Arctic states and populations. Greenlandic Inuit are disadvantaged with regard to several health indicators when compared to Danes as Greenlanders perceive for example lower lifetime expectancy rates and higher infant mortality rates and years of life lost due to suicide, while little difference is found between the Sami and non-Sami population (9, 15). Adolescent mental health care and services are relatively well staffed in Sami areas including easier access to treatment facilities, in contrast to most parts of Greenland (9).

## Limitations

There are several limitations to be noted. The dependent variable SRH was not identical as the WBYG had a fifth option *very poor*, although no Inuit reported *very poor*. Also, the NAAHS had a category *not so good*, while the corresponding category in the WBYG was worded *fair*. This could have contributed to the observed skewness in SRH. Both instruments tapping SRH were non-comparative measures, although not identical with regard to rating options (SRH-4/SRH-5), and comparisons should therefore be made with some caution. However, the current limitation is believed to only have minor influence as a Swedish study found that different non-comparative measures of SRH represents parallel assessment of subjective health (37). Conservative figures for Inuit risk behaviours may occur due to a high school absence rate. The WBYG included the word *seriously* in the question about having considered suicide, which probably led to underreporting as former suicidal thoughts may in retrospect not have been considered as “serious”. It is unknown whether the observed Sami–Inuit differences in SRH reflect true differences or cultural differences in how health-related factors were understood in the two groups (1). Potential protective cultural resilience factors such as traditional activities, spirituality, language use and healing were not included here, and these merit further research attention (38). A further limitation is that sexual abuse, parental substance use and geography, which strongly influence SRH (6), were not available for comparison here for technical reasons. A history of childhood sexual abuse has been negatively associated with SRH among Greenlandic Inuit, in particular among women and those who had experienced parental substance abuse during childhood (1). Furthermore, there are considerable geographical differences in living conditions and health in Greenland (9, 15). Our studies were cross-sectional, in line with the majority (73%) of studies on youth SRH (6). Future studies should be longitudinal and capable of assessing whether suicidal behaviour and physical inactivity temporally precede poor SRH or vice versa.

## Conclusion

Most Sami and Inuit adolescents have good SRH and do not face health hazards such as heavy drinking, exposure to violence or suicidal behaviour problems. The poorer SRH among Inuit may partly be due to group differences in risk and protective correlates associated with SRH. Physical activity was positively associated with SRH while suicidal behaviour influenced SRH negatively in both indigenous groups. The positive impact of physical activity on SRH should be targeted in future school health promotion programs.
